# Clinical Management of HIV Drug Resistance

**DOI:** 10.3390/v3040347

**Published:** 2011-04-14

**Authors:** Karoll J. Cortez, Frank Maldarelli

**Affiliations:** HIV Drug Resistance Program, NCI, NIH, Building 10 Rm 5A06, Bethesda, MD 20892, USA; E-Mail: karoll.cortez@fda.hhs.gov

**Keywords:** HIV, drug resistance testing, clinical management

## Abstract

Combination antiretroviral therapy for HIV-1 infection has resulted in profound reductions in viremia and is associated with marked improvements in morbidity and mortality. Therapy is not curative, however, and prolonged therapy is complicated by drug toxicity and the emergence of drug resistance. Management of clinical drug resistance requires in depth evaluation, and includes extensive history, physical examination and laboratory studies. Appropriate use of resistance testing provides valuable information useful in constructing regimens for treatment-experienced individuals with viremia during therapy. This review outlines the emergence of drug resistance *in vivo*, and describes clinical evaluation and therapeutic options of the individual with rebound viremia during therapy.

## 1. Introduction

Combination antiretroviral therapy has resulted in marked improvements in morbidity and mortality from HIV-1 infection [1–3]. Therapy is not curative, however, and one of the most profound limitations of current antiretroviral therapy is the development of antiviral drug resistance [4]. HIV drug resistance occurs in a substantial proportion of treated patients and accumulates over time on therapy. Although the frequency of drug resistance has declined with the introduction of better tolerated regimens, resistance is still reported in 7–15% of patients initiating first line antiretroviral therapy [5–7]. Emergence of drug resistance has consequences for individuals and populations. For individuals, drug resistance restricts subsequent antiretroviral treatment choices and can exhaust therapeutic options, resulting in HIV-1 disease progression and death. Drug-resistant variants are also transmitted when new infections occur, effectively multiplying the individual drug failures and creating a growing public health concern. Surveillance studies report that the prevalence of drug-resistant mutations among recently infected drug-naïve individuals ranges from *ca.* 5–15% [8]. As such, transmission of drug resistance threatens to reverse the reductions in morbidity and mortality accomplished by antiretroviral therapy. Emergence of drug resistance is a consequence of a combination of viral, pharmacologic, and host factors. Identification, evaluation, and treatment of HIV drug resistance represent a compelling challenge for patients and health care professionals. Management of clinical resistance is a comprehensive process that determines the cause of rebound viremia and develops a useful course designed to re-suppress HIV replication. In this review we will describe factors in the development of drug resistance, and current issues in the clinical management of HIV drug resistance *in vivo*.

## 2. Sources of Drug Resistance

Since the development of antiretroviral therapy, over 20 chemotherapeutic agents have been FDA approved. Therapy is been directed against five principal viral targets, including attachment, fusion, reverse transcription, integration, and protease mediated maturation. Regardless of drug target, resistance may emerge to any antiretroviral, and viral, pharmacologic, and host factors contribute to the emergence of drug resistance. The replication program of HIV is rapid (T_1/2_ approximately 1 day) and error prone (mutation rate *ca.* 3 × 10^−5^ mutations/base/replication cycle) resulting in large and genetically diverse populations *in vivo* from which resistance may emerge [9]. Analysis of kinetics of emergence of drug resistance *in vivo* suggested that many single nucleotide mutations conferring drug resistance might be present prior to initiation of antiretroviral therapy. Early studies demonstrating rapid emergence of the single nucleotide mutations M184I and M184V resistance to 3TC suggested that therapy represents a selective pressure permitting emergence of resistant variants [10–12]. Pre-existing resistance is strongly supported by subsequent studies demonstrating the rapid, frequent emergence of drug resistance mutations after single dose of the non-nucleoside reverse transcriptase inhibitor (NNRTI) nevirapine [13]. Direct identification of drug resistance mutations pre-therapy has also been reported using sensitive allele-specific PCR amplification capable of detecting drug resistance at levels of *ca.* 0.3% [14].

Reverse transcriptase strand transfer events occur during reverse transcription. These events result in frequent recombination and as many as 6–7 strand transfers may take place during proviral synthesis. Reverse transcription and recombination of virions containing non-identical RNA copies encoding different individual resistance profiles will result in chimeric proviral DNA molecules with concatenated individual resistance mutations [15]. As a consequence, recombination is a potent mechanism for rapid spread of drug resistance mutations within an individual. Pharmacologic factors contribute to the development of resistance. In general, antiretroviral drugs are well absorbed and generate high drug levels capable of inhibiting HIV replication. Several agents, specifically the NNRTI class, have long half lives relative to the other regimen components. During non-adherent periods, short half life agents are eliminated relatively quickly, while longer agents become essentially monotherapeutic agents, which can select for drug resistance. Individuals taking antiretroviral medications often take additional therapeutic agents for co-morbid illnesses; drug interactions may result in changes in antiretroviral drug levels [16,17]. Although ongoing therapeutic drug monitoring has not become a clinic routine [18,19], drug level testing for all FDA approved antiretroviral is available and may be useful in evaluating whether sufficient drug levels are achieved, especially in individuals taking complex multidrug regimens to treat HIV and other illnesses, where drug interaction issues may arise.

Host factors, principally drug adherence, have a strong effect on the development of drug resistance. Early studies of antiretroviral therapy demonstrated frequent emergence of resistance to antiretroviral agents; regimens were complex, required frequent dosing, and were associated with a number of adverse effects; therapy interruption was relatively common, and rapidly resulted in development of drug resistance. With newer and better tolerated combination regimens, including those with once daily dosing, adherence generally improved in individuals taking first line regimens, and drug regimen failures have declined. Nevertheless, adherence remains a central issue in the development of resistance [3,20,21]. Direct observed therapy has been useful in investigating the virologic and immunologic effects of rigorously controlled drug delivery, although questions remain regarding the degree of improvement over voluntary therapy [22] and the content of care used to address adherence can predict virologic suppression [23]. Recent studies have suggested that the effect of nonadherence is not uniform [3], and that the probability of rebound viremia with non-adherence may decrease after viral suppression is achieved. A study from the REACH cohort studied 221 patients initiating antiretroviral therapy and estimated the probability of rebound viremia for various ranges of adherence after viral suppression is achieved. The probability of virologic failure after 1 month *vs.* 12 months of continuous HIV suppression with 50–74% adherence was 0.47, and 0.36 at 90–100% adherence [24]. In independent studies, Bello and colleagues [25] investigated the level of viremia associated with long-term suppression; viremia > 100 copies/mL plasma were associated with accumulation of new genetic diversity over time while little or no evidence was detected at lower viral RNA levels. The development of once daily regimens and combination formulation of antiretrovirals represent great improvements in therapy, making HIV therapy similar to therapy for other chronic diseases requiring daily therapy, such as therapy for hypertension, diabetes, and seizure disorders. Adherence to therapy for these other illnesses also presents a number of challenges; behavioral research in these diverse areas may yield new and useful strategies to improve adherence.

With the observations that HIV persists during therapy, it has become clear that understanding the nature of HIV replication during therapy has direct bearing on the potential for the emergence of drug resistance during therapy. If active spreading infection of HIV continues during drug suppression, then the potential for new mutations and drug resistance is possible. Alternatively, if drug suppression completely blocks spreading infection, the potential for emergence of new drug resistance mutations from chronically infected, long lived reservoirs is severely limited. Seminal studies by Persaud and coworkers demonstrated no emergence of new drug resistance mutations on therapy [26], while Martinez-Picardo and coworkers did identify emergence of mutations in individuals with prior therapy and transient viremic periods [27]. Subsequently, data supporting the presence and absence of active replication during therapy have been reported, reviewed by [28]. In a set of interventional studies, drug intensification has been used as a strategy to investigate whether ongoing replication takes place during suppressive antiretroviral therapy, reviewed by Maldarelli [29]; a number of studies have detected no evidence of decreased viremia during drug intensification using sensitive single copy detection assays. Buzon and colleagues [30] have identified patients with increased levels of 2-LTR circular DNA during raltegravir intensification, suggesting that, in some patients, residual HIV replication may be present. Research to confirm these findings and to characterize patients undergoing antiretroviral therapy with complete suppression or ongoing replication continues.

Host genetic variation can have strong effects on the course of HIV infection. There are strong host encoded differences in the rate of metabolism of antiretrovirals; such pharmacogenomic issues can contribute to drug half life, and may result in underexposure leading to resistance, or over-exposure leading to toxicity of antiretrovirals [31]. In addition, specific host traits, such as HLA, have direct effect on development of cutaneous hypersensitivity to drugs including the antiretroviral abacavir [32]. Research in this areas continues to expand to include genome wide understanding of host-virus interactions.

### 2.1. Laboratory methods for detection of antiviral drug resistance

#### 2.1.1. Genotyping

Genotyping identifies resistance by a three step process of (1) nucleic acid analysis of relevant portions of the HIV genome derived from plasma, (2) identifying mutations associated with drug resistance, (3) constructing a drug resistance report. Nucleic acid analysis consists of extraction of HIV RNA from plasma, reverse transcription and PCR amplification of relevant portions of the HIV genome. HIV has broad genetic diversity that is lost, to a significant degree, during the amplification process; as a consequence only the most common variants (present in at least 15–20%) are represented in the genotypic information provided to the health care professional and the patient. Resistance associated mutations are typically “archived” in cells for apparently indefinite periods. Thus, it is possible that drug resistance may be present in the individual with rebound viremia on therapy but not detectable in resistance assays. Direct nucleic acid sequencing represents a common mechanism to obtain resistance information; commercial genotyping services, as well as systems for laboratory use are available; routine testing with independent panels of resistant viruses is useful to maintaining proficiency in detection of mutations [33–35]. Assays for specific mutations by selective hybridization are also available commercially in selected parts of the world; such “line probe” assays are reported to be more sensitive for detection of low level mutations, but are limited by the number of mutations available for assay [36–38]. It is possible that natural genetic variation surrounding the mutation site may affect detection of mutations. Mutations are identified by comparison with HIV “wild type” sequences. In the setting of wide genetic diversity “wild type” HIV is certainly only an approximation; commercially, laboratory infectious clones, such as pNL4-3 [39] or LAV [40] are used as representative viruses. Drug resistance sites have been identified *in vivo* and *in vitro. In vivo*, the emergence of specific changes on rebound viremia during therapy are identified and studied *in vitro*, demonstrating that introduction of specific mutations into wild type (WT) virus recapitulates drug resistance. *In vitro* cultivation studies also identify drug resistance mutations, typically with gradual emergence of resistance during long-term passage of HIV in the presence of antiretrovirals. The combination of *in vivo* and *in vitro* studies has yielded a comprehensive list of drug resistance mutations [35]. Commercial genotyping report provides the list of mutations conferring drug resistance, and also synthesizes the genotypic information into an interpretation provided to the health care professional, reporting predicted resistance for each drug. The predictions are either rule based, or the product of algorithms derived by analyses of large proprietary databases; all are detailed, but none are perfect [41,42]. The algorithms and rules are regularly updated with new accumulated data; new mutations may be added while others may be dropped. In addition, a number of public access sites (e.g., Stanford University HIV Drug Resistance Database [43]) are available to analyze genotypic information obtained from home-brew or commercially obtained sequences. Such sites may be useful for updated analysis of genotypes obtained 5–10 years ago that were originally interpreted with older algorithms.

In addition to reporting resistance identification, HIV drug resistance reports offer a wealth of additional useful information, particularly when combined with clinical information; five examples illustrate clinical utility of careful evaluation of genotypic data. First and most obviously, the absence of any drug resistance mutations, in the setting of high rebound viremia strongly suggests adherence issues may be the cause of rebound viremia. Second, the emergence or loss of certain drug resistance mutations responds relatively rapidly to the presence of the antiretroviral drug pressure. Resistance to 3TC or FTC is typically accompanied by the mutation M184V; M184I also confers resistance to cytidine analogues, but is usually only transiently detected, if at all, early during therapy with 3TC or FTC. M184I is rapidly outcompeted by the M184V mutation. As a result, the presence of the M184I mutation suggests that individuals have recently initiated (or re-initiated) therapy with 3TC. M184V itself responds relatively rapidly to the presence of 3TC; loss of this mutation during therapy with 3TC raises adherence concerns. Third, the presence of polymorphisms at resistance sites may be a useful observation. The presence of polymorphisms at sites strongly selected by the regimen (e.g., M41M/L, during AZT therapy) suggests that drug selection pressure has not been uniform, and non-adherence may play a role in rebound viremia. Fourth, genetic analysis and detecting the presence of uncommon mutations may offer useful insight into the origin of the infection in an individual. Genotypic analysis is used to determine HIV subtype; non-B subtypes are readily detected by this method, and can provide useful information regarding geographic source of infection. Uncommon mutations at position 215 of reverse transcriptase, such as T215S, T215C, reflect back mutations [44], suggest the prior presence of T215Y or F, and often represent strong evidence of transmitted drug resistance. Finally, some resistance sites are reported to confer resistance to one antiretroviral and may confer increased sensitivity to other antiretroviral drugs. Examples include K65R, which confers high level resistance to tenofovir, confers increased sensitivity to AZT; the mutation T215Y confers resistance to AZT, but is more sensitive to tenofovir than the wild type T215. Clinical correlates of these *in vitro* observations are uncertain but may be useful in constructing new regimens to treat the resistant virus [45].

#### 2.1.2. Phenotyping

**Figure 1.**
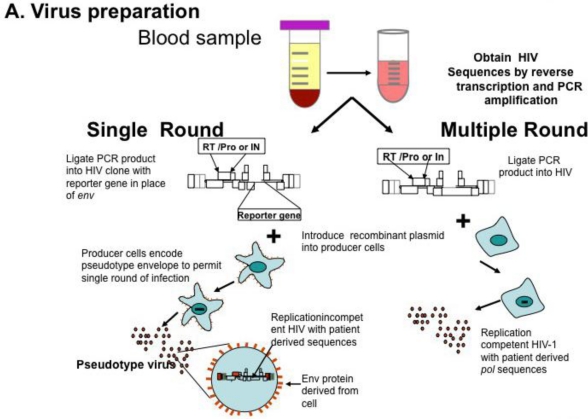

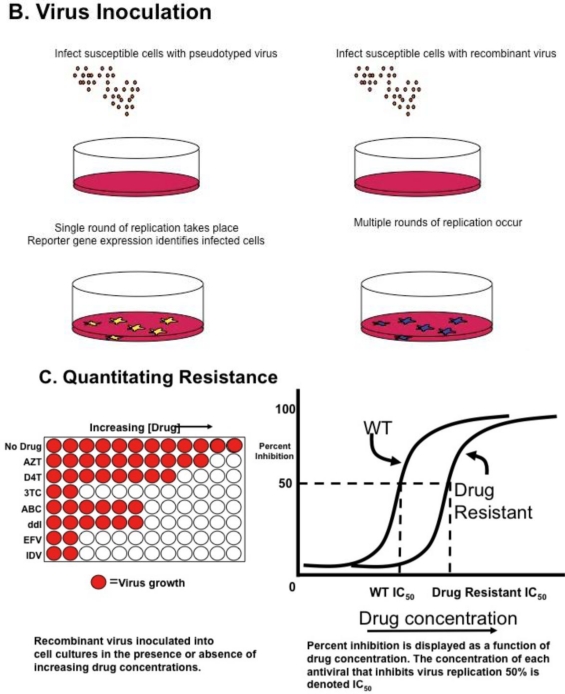
HIV Phenotyping of HIV protease (PRO), RT, integrase (IN) in Cell Based Assays. (**A**) Schematic of sample processing from phlebotomy to construction of chimeric recombinant plasmids composed of patient derived sequences in standard laboratory based HIV standard clones. Single round assays use HIV derivatives encoding a reporter gene instead of HIV *env.* Upon transfection into producer cells expressing a helper virus envelope, virions are produced which can undergo a single round of replication. Multiple round assays introduce patient-derived material into standard laboratory HIV, and recombinant plasmids transfected into producer cells. (**B**) Virions produced by transfection are standardized and used to infect susceptible cells. In single round assays, pseudo typed viruses undergo reverse transcription and integration, but are unable to propagate. Production of reporter gene product (e.g., luciferase, green fluorescent protein) denotes successful round of replication. In multiple round infections, virus is inoculated and production measured by standardized measures, typically production of p24 antigen in media. (**C**) To determine phenotypic response to antivirals, virus is inoculated in the presence of increasing concentrations of single antiretrovirals. Dose response curves are constructed and measure of drug inhibition; the amount of drug necessary to inhibit 50% of virus replication (IC_50_) is calculated. Adapted from [68].

Genotyping information may yield complex mutational patterns. Phenotyping assays have been developed in an effort to provide a functional evaluation of patient derived HIV-1 protease and reverse transcriptase. Recombinant DNA technology [46] generating chimeric plasmids with gag/pol sequences from patient isolates cloned into laboratory-adapted HIV-1 strains permits reliable and reproducible measurement of *in vitro* resistance; several assays are commonly available using different strategies as single round or multiple round infections [35,47,48] (Figure 1A). HIV-1 protease, RT, integrase, and envelope sequences amplified from patient material using RT-PCR techniques as described above for genotyping are introduced into a recombinant molecular clone of HIV-1, either by direct ligation, or by simply mixing and allowing ligation to occur during the transfection [49]. Virions produced by transfection are standardized and used to inoculate cultures of susceptible cells (Figure 1B); in parallel, cultures are inoculated with wild type virus. Infections are carried out in the presence of increasing concentrations of individual antiviral agents, viral replication measured and dose response curves constructed (see Figure 1C); the concentration of drug necessary for 50% inhibition of virus replication is reported as IC_50_. Conceptually, the two approaches appear to offer different advantages. Single round assays eliminate the possibility that resistance may arise during the cultivation of wild type virus in the presence of the drug [50]. Multi-round cultivation assays may detect low level resistance that may not manifest in a single round assay. Direct comparison of these two assays revealed remarkably concordant results [51].

Both phenotyping systems evaluate only a portion of patient derived material; any interactions between portions of HIV genome from patient derived virus are not measured. For example, phenotypic analysis of patient derived HIV protease takes place without inclusion of most protease cleavage sites as additional compensatory changes may occur at cleavage sites, it is likely that a degree of phenotypic information is lost in the process [52–54]. New phenotypic assays have been developed to investigate HIV tropism to evaluate patients who are under consideration for therapy with coreceptor inhibitors. Such assays are can detect the presence of X4 or dual X4/R5 tropic virus at low levels and are recommended prior to drug initiation, as the presence of X4 or dualtropic virus is likely not to be susceptible to coreceptor inhibitors in viremic patients [35].

Increases in IC_50_ are associated with drug resistance, but the degree of virologic resistance associated with clinical drug failure is not clear in all circumstances. Establishment of effective cutoffs has been a major effort in phenotyping development. For some antiretrovirals, such as efavirenz or 3TC, large increases in IC_50_ are noted, and identifying resistance is straightforward. In contrast, drugs such as ddI or d4T have a more restricted dynamic range in phenotyping assays. Phenotypic sensitivity scores were predictive of viral RNA responses in treatment experienced patients initiating protease inhibitor regimens [55], demonstrating the utility of phenotypic results and RNA responses, but precise cutoffs are relatively difficult to assign. One approach has been to evaluate the distribution of drug resistance in isolates from drug naïve individuals and assigning cutoffs at IC_50_ levels several standard deviations beyond the mean drug naïve level [56]. Use of clinical trial and cohort analysis has also been proposed to estimate clinical cutoffs [57]. Increasing knowledge regarding the nature of drug resistance and the success of commercial drug resistance testing has permitted analysis of large numbers of matched HIV genotypes and phenotypes and development of bioinformatics tools that predict phenotypic responses based solely on genotypic information. These analyses have been studied in clinical trials and are commercially available [58–60] or open access [61] e.g., [62]or ANRS [63] and Rega algorithms [64] and the Stanford HIVDB [43] are generally well correlated with experimental phenotypic drug resistance data and have recently been reviewed [65,66]. A current list of resistance mutations is maintained and updated regularly [67].

Direct comparisons of virtual phenotypic data with genotype and phenotype have not been extensively investigated. In the CREST trial, Emrey and colleagues [69] did not detect a difference in virologic success in a randomized study of individuals undergoing drug resistance testing with or without virtual phenotyping; Saracino and coworkers obtained similar virologic suppression in patients using virtual or real phenotypic results [59]. A number of research approaches have incorporated IC_50_ phenotypic data with levels of drug measured in individual patients. These inhibitory quotients have correlated well with drug suppression [70,71], but have not been used extensively yet in routine clinical practice.

An entirely new analysis of phenotypic data derives new and useful information from slopes of the dose response curve [72] used to determine instantaneous inhibitory potentials. Dose response slopes were found to be class specific, and these authors established limits for the inhibitory activity of individual drugs and for entire classes. In this analysis, NNRTIs and protease inhibitors had the greatest activity against wild type virus. The performance characteristics of the instantaneous inhibitory potential have not yet been field tested in detail or with drug resistant virus. An initial analysis by Kuritzkes and colleagues obtained similar outcome data using IIP or IQ, and the clinical utility of these modalities remains under study [73].

## 3. Clinical Utility of Drug Resistance Testing

### 3.1. Evidence Base for the Use of Drug Resistance Assays in Clinical Management of HIV-1 Infection

A number of randomized, prospective studies investigated the utility of resistance testing in management of antiretrovirals [74–78]. VIRADAPT [79] and GART [80] were two early prospective genotyping studies that randomized patients to two arms: genotyping and standard of care. The benefit in both of these trials appeared to be the ability of genotyping to identify a greater number of active antiretrovirals. VIRA3001 [75] was a randomized study comparing phenotyping and standard of care in 272 viremic patients; at the week 16 endpoint phenotyping arm had demonstrated a greater effect on reducing HIV-1 viremia measured by proportion of patients <400 c/mL; in the secondary analysis, phenotyping was also superior to SOC in reducing viremia, measured by area under the curve minus baseline. The specific benefit of additional “expert advice” e.g., panels of investigators with extensive experience in HIV therapy was specifically investigated in HAVANA [78], a factorial design study comparing both the use of genotyping and the use of expert advice in virologic outcome (VL < 400 c/mL). Genotyping was superior to no genotyping even in the absence of expert advice, using only the algorithm interpretation of the genotype. In addition, expert advice was beneficial even in the absence of genotyping; more recently, higher agreement rates among those with “expert opinion” suggests more consensus among therapeutic options [81].

Duration of these studies were relatively short; ARGENTA was a prospective randomized study comparing efficacy of genotyping and standard of care at two time points [76] in reducing viral loads. Although the proportion of patients suppressed <500 c/mL was significantly greater in the genotyping arm after 12 weeks, the effect was not sustained and no benefit of genotyping was detected at 24 weeks. The durability of resistance testing beyond one year has not been studied prospectively. Many of the studies were performed prior to widespread use of sensitive <50 copy limit assays and used <400 c/mL or <500 c/mL as a measure of success; it is not clear whether all successful treatments reached the more stringent measure of suppression, but the virologic benefit realized in genotyping arm of the ARGENTA was persistent in subsequent longitudinal analysis [76].

Several trials have not demonstrated benefit of resistance testing. NARVAL was a randomized study of phenotyping, genotyping and standard of care [77] no benefit of resistance testing was noted in the entire cohort; in a sub analysis of patients a benefit of genotyping in patients with a single prior PI-containing regimen was detected. NARVAL patients were in general more drug experienced than in prior trials, and the absence of effective drugs regardless of resistance testing may have contributed to the equal success rates. NARVAL was initiated after resistance testing had been generally introduced, and physicians had the benefit of earlier trials of the performance of antivirals in various drug resistance settings.

In these early studies, participants were generally less antiviral experienced than patients currently failing therapy. The clinical benefit of resistance testing in viral suppression in highly experienced patients has not been extensively investigated. In NARVAL [77], no significant differences in adverse event reporting were detected in resistance testing and standard of care arms. The CCTG575 Study of 238 patients with drug resistance did not demonstrate a clear benefit of phenotyping over standard of care, although benefits were noted in patients with more resistant virus [82]. A meta analysis of the effectiveness of drug resistance testing of ten trials highlighted the short term nature of the benefits of genotyping and virtual phenotyping [83]. In contrast, retrospective analysis of 2699 of the HOPS natural history cohort study patients over the period 1999–2005 revealed a survival advantage in management of drug resistance that included drug resistance testing compared with expert advice alone [84]. Drug resistance testing has become part of the standard of care of individuals with rebound viremia and remains recommended by guidelines for treatment of HIV infected individuals [85]. Resistance testing is recommended in the initial evaluation of HIV–infected individuals at the time of diagnosis, in order to identify individuals with transmitted drug resistance and at confirmed rebound in viremia [85,86].

## 4. Drug Class Specific Issues

### 4.1. NRTI

**Figure 2.**
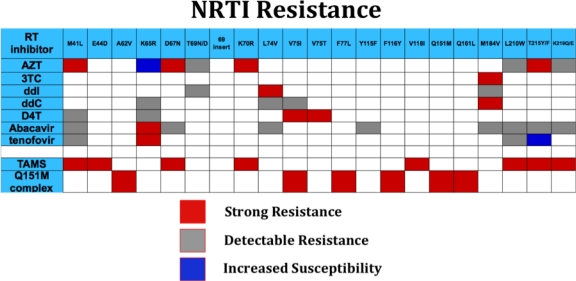
Mutations conferring resistance to nucleotide reverse transcriptase inhibitors (NRTIs) are depicted; multidrug resistance profile (Q151 complex) is indicated and thymidine associated mutations (TAMS) are noted.

Reverse transcriptase is the central enzyme in HIV replication and mediates RNA-dependent DNA synthesis, RNase H excision of HIV RNA from RNA: DNA hybrid, and DNA directed DNA synthesis. A reverse, “excision” reaction, which removes incorporated nucleotides, also occurs at a low rate compared to polymerization. RT assembles as a dimer of p66 and p51 subunits; like a number of DNA polymerases, the structure of RT is similar to a right hand, and includes a palm, fingers, and thumb domains [87]. Nucleoside and nucleotide reverse transcriptase inhibitors (NRTIs) represent strong inhibitors of reverse transcriptase and inhibit polymerization by chain termination. NRTIs such as AZT, 3TC, and ddI represent some of the oldest antiretrovirals, and many individuals with long antiretroviral experience have had extensive exposure to NRTIs singly or in combination, with accumulation of a number of drug resistance mutations (Figure 2). In general, mutations, result in steric effects on drug access, e.g., M184V, 3TC, ddC [88], or kinetic effects that result in marked increase in excision of incorporated analogues [89]; thymidine associated mutations (TAMs, including M41L, D67N, K70R, L210W, T215Y/F, K219Q) confer resistance by enhanced excision. TAMs excision may be reduced by M184V. In addition, M184V-containing virus is less fit than wild type virus [90,91], perhaps providing an additional virologic benefit. Accumulation of TAMS results in increasing resistance to AZT, tenofovir, D4T, abacavir, and ddI. Two multidrug resistance mutation profiles in general, confer high level drug resistance to all NRTIs. The Q151M suite of mutations (Figure 2) results in selective decreased binding of NRTI triphosphate and a degree of increased excision [92,93] ; although tenofovir has some reported activity in this setting [94], while other NRTIs such as AZT, D4T, ddI, and abacavir are uniformly ineffective. Insertions following the threonine residue at position 69, typically consisting of 1–4 additional residues result in uniformly high level resistance to all NRTIs.

Accumulation of drug resistance mutations reduces the rate of reverse transcription. RNase H activity of RT, which removes RNA from the RNA:DNA product of first round reverse transcription must work in concert with polymerization. Pathak and coworkers hypothesized that additional mutations affecting RNase H activity may compensate for accumulated mutations slowing RT mediated polymerization [95]. Such resistance mutations have been identified in the connection domain of reverse transcriptase [95–97]. Although this region of RT is not typically included in commercial genotyping, connection domain mutations may increase resistance to certain NRTIs, such as AZT, by 10–100 fold [35,96,97]. The clinical consequences of these new NRTI resistance mutations remain under intense study.

### 4.2. NNRTI

**Figure 3.**
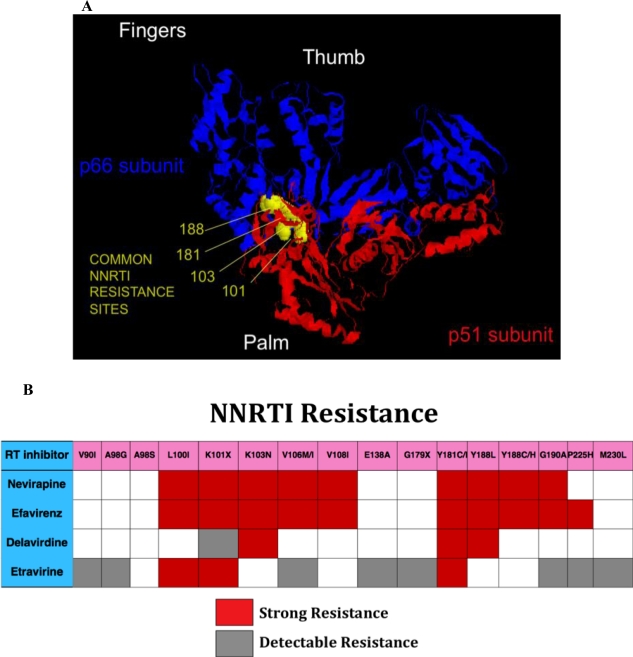
(**A**) Crystal structure of HIV reverse transcriptase p66/p561 dimer is depicted with locations of four common NNRTI resistance mutations in a hydrophobic pocket within the palm domain noted in yellow. Mutations change the binding characteristics of NNRTI, explaining cross resistance of nevirapine, delavirdine, and efavirenz. Structural data are from [99] and are displayed using RASMOL [100,101]. (**B**) Mutations conferring resistance to individual NNRTIs. Despite some cross resistance, etravirine has antiviral activity even in the presence of a number of mutations conferring resistance to nevirapine and efavirenz.

NNRTIs bind to a common hydrophobic pocket in the palm domain of RT that is near, but not at the HIV RT catalytic site (Figure 3A). Mutations conferring resistance result in changes in binding characteristics for the inhibitors [98]. In general, there is extensive cross-resistance among NNRTIs (Figure 3B). The majority of NNRTI resistance mutations do not significantly reduce the replication capacity of HIV, and tend to persist for prolonged periods. The newest NNRTI, etravirine, has significant antiviral activity even in the setting of a number of NNRTI resistance mutations, including K103N and Y188C. As a result it is possible to construct NNRTI-based regimens in individuals with NNRTI resistance. One challenging aspect of such a strategy is ensuring that etravirine mutations (Y181C) are not present in individuals, some of whom may have remotely taken NNRTI. It is not clear that relying on population-based based genotypes, which only report on contemporary and predominant HIV sequences, will provide sufficient sensitivity to rule out the presence of concerning mutations.

Several studies have demonstrated new RT mutations accumulating on NRTI that confer increased susceptibility to reverse transcriptase [102–104]. The mechanism by which new mutations confer hypersusceptibility to NNRTI is uncertain. Hypersusceptibility may affect nucleotide selectivity or virus replication capacity [105,106]. Clinical advantage of NNRTI hypersusceptibility has not been extensively investigated, but patients with hypersusceptibility did experience higher reductions in viral RNA, suggesting a clinical advantage [103,107].

### 4.3. Protease

**Figure 4.**
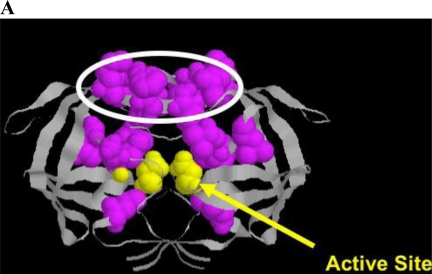

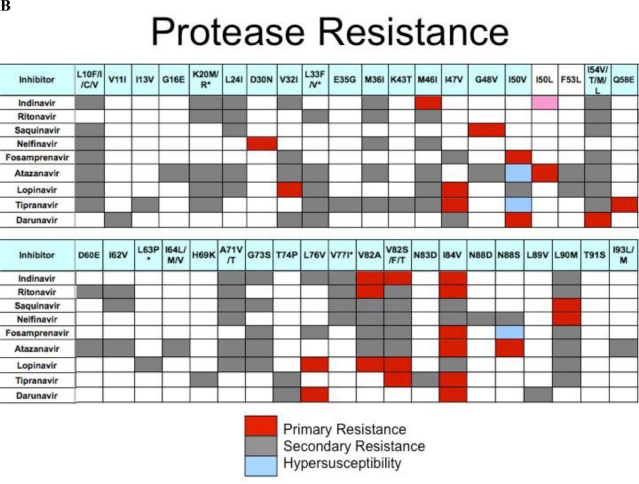
(**A**) Crystal structure of HIV protease depicting active site residues (yellow) and a series of residues conferring resistance near the active site or at flap domains (circled). Structural data are from [114] and are displayed using RASMOL [100,101]. (**B**) Chart depicting resistance to HIV-1 protease.

HIV protease processes the Gag and Gag/Pol protein precursors, resulting in virus maturation and virus infectivity. Protease inhibitors, either peptidometic or nonpeptidometic, are designed to bind to the active site of the protease. Protease is only 99 amino acids in length, but tolerates a relatively large number of mutations that confer resistance (Figure 4A). A substrate fit model developed by Schiffer and colleagues has developed a useful model explaining drug resistance mutations that emerge near the active site and in flap domains of the molecule that provide access to the active site [108–110]. In general, mutations conferring resistance (Figure 4B) are divided into primary mutations, generally drug specific, and secondary mutations, which by themselves confer little resistance to therapy, but in the presence of primary mutations result in increased cross resistance to a number of protease inhibitors [111,112]. Thus, switching protease inhibitors carries a measure of phenotypic “baggage” that can affect subsequent protease inhibitor therapy. Additional mutations occur at protease cleavage sites, resulting in increased efficiency of cleavage despite accumulation of changes in the enzyme proper. Ritonavir is a potent inhibitor of the cyp3A4 metabolic pathway metabolizing many protease inhibitors, and the use of ritonavir to pharmacologically boost protease inhibitor levels in patients has been a useful strategy to maintain protease inhibitor levels and permit once daily dosing. New agents with similar activity are under consideration, including one in clinical trials [113].

### 4.4. Coreceptor Inhibitors

Maraviroc inhibits binding of HIV gp120 to the CCR5 coreceptor of HIV. As HIV variants may utilize either CCR5 or CXCR4 (or both, “dualtropic” viruses) as coreceptor, resistance to Maraviroc may occur as a population shift to CXCR4-tropic or dual tropic viruses. Sensitive tropism assays are available to determine presence of CXCR4 or dual tropic viruses at relatively low frequencies (reported at 0.1%) [115]. Such tropism assays are required prior to initiating therapy, as the presence of dual tropic or CXCR4 tropic virus compromises Maraviroc efficacy. In addition, resistance can occur by emergence of mutations that affect binding of Maraviroc. Thus, the etiology of rebound viremia on Maraviroc or other coreceptor inhibitors may be multifactorial.

### 4.5. Fusion Inhibitors

Enfuvirtide [116] is a 36 amino acid peptide that binds to alpha helix within gp41, disrupting the “spring-loaded” like mechanism that mediates viral-cell fusion [116,117]. Early studies identified mutations emerging in the binding domain abrogating enfuvirtide activity, and found such changes in great majority of patients with rebound viremia while adherent on enfuvirtide therapy [118,119]. Thus it is typically not cost effective to genotype rebound viremia for T-20 resistance mutations. Deeks and coworkers demonstrated continued T-20 antiviral activity even in the presence of mutations [120]. It is likely that drug resistance mutations reduce replication capacity; the potential clinical benefit of maintaining T-20 despite resistance is weighed against the inconvenience, discomfort, and relative difficulty in twice daily subcutaneous administration.

### 4.6. Integrase Inhibitors

**Figure 5.**
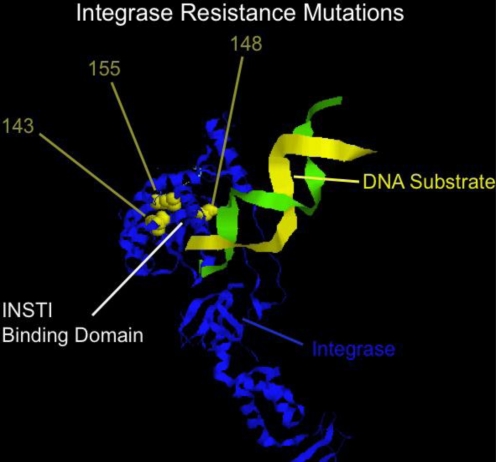
Crystal structure of human foamy virus integrase (similar to HIV integrase) complexed with DNA substrate, noting the positions of resistance mutations 143, 148, and 155 relative to the binding site for raltegravir and elvitegravir, and proximity to DNA. Structure is from Cherapenov and coworkers [128] and rendered in RASMOL [100,101].

Raltegravir is the first in class integrase strand transfer inhibitors (INSTI) and represents a highly potent and well tolerated addition to the antiviral armamentarium [121–123]. A second INSTI, elvitegravir, is currently in late stage development [124]. Despite the marked activity and ease of tolerability, raltegravir is, however, similar to all antiretrovirals in that resistance can emerge relatively quickly if the drug is used in combinations with ineffective or recycled antivirals. A number of independent pathways to resistance have been identified, with mutations at positions N155H, Q148H/R/K, or Y143R/H/C yielding high level resistance to therapy, typically with a number of additional secondary mutations [125]. Mutations map near the binding site of the inhibitor (Figure 5), and a common binding site for raltegravir and elvitegravir explains, in part, the cross-resistance between these two inhibitors [126]. Additional INSTI are in development, at least one of which has reported to have some evidence of non-cross resistance to raltegravir [127]. Phenotyping for INSTI are now commercially available.

## 5. Non-Subtype B and HIV-2 Infections

### 5.1. Non-Subtype B Infection

Although the vast majority of genotypic information has been obtained using HIV group M, subtype B, investigations of non-B viruses are ongoing and increasing [129,130]. A number of differences in drug resistance have been reported [131]; for example the rate in which certain mutations occur, e.g., K65R appears to occur in subtype C faster than in subtype B [130], and the NNRTI drug resistance mutation V106M is commonly identified in subtype C but not B [132,133]; the emergence of either mutation may be explained, in part by the baseline wild- type sequence favoring the development of the mutation. In general, however, non-B subtypes have similar sensitivities and responses to antiretrovirals as subtype B [134–138]. Non-M viruses have not been extensively studied, although group O viruses are intrinsically resistant to the NNRTI nevirapine and efavirenz. Initial data on etravirine [139] and raltegravir [140] resistance in non-B viruses has been reported.

### 5.2. HIV-2 Infection

HIV-2 infection shows striking differences in drug sensitivity from HIV-1. Although some variability among isolates exists, HIV-2 is intrinsically resistant to NNRTIs; susceptibility to etravirine has not been studied extensively. NRTI resistance emerges in HIV-2, and has a higher prevalence of multidrug resistance Q151M suite of mutations (A62V, V75I, F77L, F116Y, Q151M) [141,142].

## 6. Clinical Management of Drug Resistance

Clinical management of HIV drug resistance proceeds as with other clinical diseases, and begins with a comprehensive history, followed by physical examination and indicated laboratory studies. In general, patients undergoing successful combination antiretroviral therapy have viral RNA levels suppressed below the commercial limit of detection (50–75 copies HIV-1 RNA/mL plasma) using either bDNA or RT-PCR based methodologies.

The first indication that drug resistance may be present is an increase in plasma viremia above the clinical cutoff during a routine clinical visit. The turnaround time for HIV RNA levels determination is at least several days, and results from testing will not be available if the clinical visit is timed with the phlebotomy for HIV RNA. One approach to this delay from phlebotomy to result is to obtain blood for viral RNA levels, and CD4 cell numbers one week prior to the clinical visit, at which time the clinical evaluation with all relevant laboratories studies may be obtained. In this way, the patient with rebound viremia may be counseled in person from the outset.

All increases in viremia should be evaluated as soon as possible, but not all increases in viremia represent drug resistance and other causes are typically ruled out prior to in depth evaluation for resistance, and repeat viral RNA testing is essential. Low level increases in viremia (blips, viral RNA 50-*ca.* 200 copies HIV RNA/mL plasma) may be the result of assay variation, and do not often reflect true rebound viremia [143]. Recent development of a new version of the Abbott amplicor PCR increases in viral RNA have been noted in the 74–200 copy range [144–146]. These increases have not been sustained or associated with resistance [147] and may reflect statistical variation near the limit of detection. Intercurrent, typically febrile, illnesses represent a second cause of rebound viremia. The precise cause of increased viremia remains uncertain, as not all illnesses result in elevations in viremia; rebounds that do occur are not associated with drug resistance, and may be the result of generalized immune activation. A third cause of rebound viremia not due to drug resistance is antiretroviral nonadherence. A thorough history and physical examination, to rule out antecedent illness or nonadherence coupled with repeat viral RNA testing represents the initial evaluation of rebound viremia.

Sustained increases in HIV viremia in the absence of other potential causes suggests that resistance mutations conferring drug resistance have emerged. Low level rebound viremia typically continues to increase, which prompts resistance testing. A minimum of viremia is essential for resistance testing; there are no absolute limits although levels *ca.* 500 copies/mL may be unreliably amplified, and tropism testing requires viremia in excess of 1000 copies/mL plasma. It is difficult to predict which component of drug regimen resistance may have failed during rebound viremia. In general, resistance mutations that confer high level resistance (e.g., K103N, efavirenz or M184V, 3TC/FTC) are likely to be present during rebound viremia. Resistance to other antiretrovirals is more difficult to predict. In addition, as described above, understanding of specific drug resistance mutations that emerge on therapy to specific agents may provide some virologic benefit in the setting of other agents. For instance, development of M184V during 3TC/FTC therapy results in a less fit virus, and one which, in the presence of TAMS, is more sensitive to NRTI therapy with AZT, D4T, or tenofovir. Similarly, K65R-containing virus arising from from tenofovir, abacavir or ddI is more sensitive to AZT than the wild type virus [45,148], T215Y/F-containing virus from AZT therapy is more sensitive to tenofovir than wild type virus. As a consequence, 3TC or FTC is often continued in individuals with M184V because of the potential antiviral effect. Tenofovir and AZT have been used in treatment experienced individuals to suppress T215Y/F and K65R. The results of these laboratory tests represent useful data, but are not themselves designed to be clinical management tools. Choice of therapy for treatment experienced individuals represents a cooperative interaction between patients and health care professionals and requires assessment of resistance, drug tolerability, adverse effect profile of potential agents, and drug interactions. Regimens are often constructed around development of newly developed agents directed against novel viral targets, but without additional active agents, the new regimen is effectively monotherapy; viral suppression is typically transient and efficacy from the new drug may be completely lost. In general, two new agents to which the patient does not have resistance are used for therapy for experienced patients [85]. With extensive cross resistance among drug classes, the goal of reducing viremia to <50 copies/mL plasma with two fully active agents, is often not possible. Despite adverse effects, partially active therapy is clearly superior to drug interruptions; useful guidelines are available to assist antiretroviral choice [85,86].

As described, therapy for HIV infection is individualized based on firm basic and clinical science. Guidelines for the use of antiretroviral therapy have been developed and are regularly updated; panels of experts review and debate accumulated evidence and provide recommendations regarding use of antiretrovirals and resistance testing. Several commendable aspects of this process include: Graded recommendations, e.g., critical recommendations well founded by randomized controlled trials are weighted with high confidence. Recommendations that are based on “expert opinion” are marked with lower confidence. In addition, some guidelines include a highlighted discussion of new or changed recommendations, providing knowledgeable practitioners with a rapid mechanism to update their fund of knowledge. The US Department of Health and Human Services guidelines is regularly updated in a “living-document” fashion [149]. As a consequence, providers worldwide have access to current and useful information.

## 7. Unresolved Issues

Although several clear instances of pre-existing mutations contributes to emergence of drug resistance, not all resistance is clearly explained by pre-existing resistance mutations, and the role of low level drug resistance mutations remains undefined. Genetic composition of HIV from drug resistant individuals is complex but can be investigated with sensitive techniques [13,14,150–153]. In one sense, since most of single drug resistance mutations are likely present in large genetically diverse virus populations [10,154], the real question is what level of resistance is associated with clinical resistance [155]. Cross-sectional studies of low level transmitted drug resistance was associated with regimen failure [156,157], and recent analysis of ACTG5095 [158] demonstrated increased risk of regimen failure in the presence of low level NNRTI mutations, and analysis of ACTG5208, randomized study of combination therapy for individuals with prior nevirapine exposure revealed relatively low level resistance [159] was associated with risk of viral rebound or death [160]. Application of new techniques, such as massively parallel pyrosequencing, may shed new light on the role of minority resistance or tropism variants in emergence of HIV drug resistance [161–164].

Viral rebound or viral suppression may occur [165] in the presence of a partially suppressive regimen. For instance, patients taking NNRTI based regimens may experience viral rebound; analysis of drug resistance may reveal emergence of a single nucleotide change conferring drug resistance [11]. Continued suppression; however, may also occur in the presence of a single mutation such as K103N. Why viremia rebounds in some patients but not others with known mutations is unknown.

The relative contributions of individual antiretroviral during rebound viremia remains uncertain, but such data might be most useful in constructing new regimens. Whether some drugs (e.g., tenofovir, protease inhibitors, INSTI) continue to have efficacy in the setting of accumulated resistance mutations has not been well described. Contributions of HIV fitness to viral replication and pathogenesis, reviewed in [166] are clear. Sensitive viral fitness assays are useful in understanding the effects of mutations on viral replication and pathogenesis [167–170], and in understanding replication of viruses from individuals [171] but are not feasible for routine clinical use. Relative replication capacity measurements are made in some phenotyping assays. When fully suppressive regimens are not possible, choosing regimens yielding lowest replication capacity may be desirable; in early studies, higher CD4 cell numbers were associated with lower replication capacity [172,173], and accumulation of drug resistance mutations is associated with lower replication capacity [125,173,174] but clear evidence for clinical benefit of lower relative replication capacity remains under study.

New bioinformatics approaches to analyze genotypic and phenotypic data continue to be investigated [72]. In general, resistance testing analyzes individual drugs singly, not in combination. New artificial neural networks are in development that incorporate resistance data with clinical information to identify entire regimens in a patient specific manner [175–177]. The use of induction-maintenance therapeutic strategies has resulted in persistent suppression for prolonged periods [159,178,179], but how a single agent maintains suppression in a substantial proportion of patients remains uncertain. Continued studies of population dynamics and the potential contribution of immune responses are likely to shed new light on the nature of drug resistance *in vivo*.
